# Long Noncoding RNA SNHG4 Attenuates the Injury of Myocardial Infarction via Regulating miR-148b-3p/DUSP1 Axis

**DOI:** 10.1155/2022/1652315

**Published:** 2022-12-05

**Authors:** Sheng Wang, Zhaoyun Cheng, Xianjie Chen, Guoqing Lu, Xiliang Zhu, Gaojun Xu

**Affiliations:** Department of Cardiovascular Surgery, Fuwai Central China Cardiovascular Hospital, Heart Center of Henan Provincial People's Hospital, No. 1, Fuwai Road, Zhengzhou City, Henan Province 451464, China

## Abstract

**Objective:**

Long noncoding RNAs (lncRNAs), including some members of small nucleolar RNA host gene (SNHG), are important regulators in myocardial injury, while the role of SNHG4 in myocardial infarction (MI) is rarely known. This study is aimed at exploring the regulatory role and mechanisms of SNHG4 on MI.

**Methods:**

Cellular and rat models of MI were established. The expression of relating genes was measured by qRT-PCR and/or western blot. *In vitro,* cell viability was detected by MTT assay, and cell apoptosis was assessed by caspase-3 level, Bax/Bcl-2 expression, and/or flow cytometry. The inflammation was evaluated by TNF-*α*, IL-1*β*, and IL-6 levels. The myocardial injury in MI rats was evaluated by echocardiography, TTC/HE/MASSON/TUNEL staining, and immunohistochemistry (Ki67). DLR assay was performed to confirm the target relationships.

**Results:**

SNHG4 was downregulated in hypoxia-induced H9c2 cells and MI rats, and its overexpression enhanced cell viability and inhibited cell apoptosis and inflammation both *in vitro* and *in vivo*. SNHG4 overexpression also decreased infarct and fibrosis areas, relieved pathological changes, and improved heart function in MI rats. In addition, miR-148b-3p was an action target of SNHG4, and its silencing exhibited consistent results with SNHG4 overexpression *in vitro*. DUSP1 was a target of miR-148b-3p, which inhibited the apoptosis of hypoxia-induced H9c2 cells. Both miR-148b-3p overexpression and DUSP1 silencing weakened the effects of SNHG4 overexpression on protecting H9c2 cells against hypoxia.

**Conclusions:**

Overexpression of SNHG4 relieved MI through regulating miR-148b-3p/DUSP1, providing potential therapeutic targets.

## 1. Introduction

Myocardial infarction (MI) is a fatal disorder with a high risk of mortality due to no obvious warning and late diagnosis [1, 2]. There are approximately 350 million people suffer from MI each year [1]. In recent years, the main therapeutic strategy for MI is reperfusion for ischemic myocardial tissues in time. However, restoration of the blood flow can induce adverse microstructural destruction and inflammation [3]. The secondary injury of reperfusion directly contributes to the poor survival of patients with MI [4, 5]. Therefore, exploring of potential therapeutic strategy with less adverse effects for MI is urgently needed.

With the development of molecular biology, more and more molecular targets exhibit therapeutic potential against MI [2]. Long noncoding RNAs (lncRNAs) are critical regulators in the pathogenesis of MI through posttranscription and chromatin modification [6]. For example, overexpression of CARL represses the apoptosis of cardiomyocytes in a rat model of MI [3]. The upregulation of Gm2691 and H19 improves heart function and relieves inflammation in MI rats [4, 5]. Small nucleolar RNA host gene (SNHG) is a group of lncRNAs that also participates in myocardial injury. Lu et al. have shown that overexpression of SNHG12 weakened hypoxia-reoxygenation (H/R) injury via inhibiting RAGE expression in H9c2 cells [7]. Xiong et al. have revealed that SNHG15 may be a potential apoptotic biomarker in myocardial cells in response to ischemia/reperfusion (I/R) injury [8]. Notably, SNHG4 also exerts a critical role in many inflammatory disorders, including proteinuria [9], neuropathic pain [10], and cerebral I/R injury [11]. However, the regulatory role of SNHG4 in MI is relatively reported.

lncRNAs can block the functional interaction between miRNAs and related target mRNAs by acting as miRNA sponges [12]. Recently, massive miRNAs have been determined as the targets of SNHG4 in cancers, such as miR-138 [13], -590-3p [9], -377-3p [14], -204-5p [15], and -224-3p [16]. There are also many miRNAs involved in MI progression. For example, miR-375 acts a pathogenic miRNA in MI through regulating myocardial dysfunction [17]. Downregulation of miR-327 decreases the infarct area of myocardial tissues in MI mice [18]. Both miR-154 [17] and miR-21 [19] function in the acceleration of cardiac fibrosis. In addition, miR-148b is also an important regulator in myocardial injury. Sun et al. have found that the downregulation of miR-148b decreases the apoptosis of H/R-induced myocardial cells [18]. Yang et al. have revealed that silencing of miR-148b-3p relieves myocardial I/R injury through enhancing antioxidation and antiapoptosis [20]. However, the function of miR-148b-3p on MI and related regulatory relationship with SNHG4 remain unclear.

The translation or degradation of target mRNAs is the main regulatory outcomes of lncRNAs/miRNAs in cardiovascular diseases [19]. There are many lncRNA/miRNA/mRNA regulatory axes that have been determined in MI, such as LINC00664/miR-197-3p/JAK2 [21], MALAT1/miR-30a/BECN1 [22], GAS5/miR-525-5p/CALM2 [23], and PCFL/miR-378/GRB [24]. Dual specificity phosphatase 1 (DUSP1) is an antiapoptotic phosphatase that is highly expressed in normal human organ tissues, such as liver, heart, and lung [23]. Candas et al. have shown that the silencing of DUSP1 acts as a suppressor in tumor growth through promoting apoptosis [25]. Tan et al. have found that the downregulation of DUSP1 contributes the improvement of cardiac I/R injury [26]. DUSP1 is also the target of many lncRNA/miRNA axes in diverse diseases, including JHDM1D-AS1/miR-101-3p in brachial plexus injury [27], GAS5/miR-429 in acute lung injury [28], and C5orf66-AS1/miR-127-3p in hepatocellular carcinoma [29]. However, the lncRNA/miRNA/mRNA axes involving SNHG4 are still not revealed in MI.

In this research, the function of SNHG4 was investigated in both hypoxia-induced H9c2 cells and a rat model of MI. The regulatory mechanisms of SNHG4/miR-148b-3p/DUSP1 axis in MI were further evaluated *in vitro*. Our study is aimed at revealing potential therapeutic targets for MI.

## 2. Materials and Methods

### 2.1. Cell Culturing and Treatments

A rat myocardial cell line H9c2 (BioVector, Beijing, China) was cultured in Dulbecco's Modified Eagle's Medium with 10% fetal bovine serum (Invitrogen, Carlsbad, CA, USA). Hypoxia model *in vitro* was established by incubating H9c2 cells in an incubator filled with 93% N_2_, 2% O_2_, and 5% CO_2_ for 24 h (hypoxia group). Cells cultured in a normal condition (74% N_2_, 21% O_2_, and 5% CO_2_) were enrolled as the control group [30, 31]. In addition, shRNA targeting DUSP1 (sh-DUSP1), sh-SNHG4, sh-negative control (sh-NC) (Transheep, Shanghai, China), miR-148b-3p mimics, miR-NC, miR-148b-3p inhibitor, inhibitor NC, pcDNA3.1-SNHG4 (pcDNA-SNHG4), pcDNA-DUSP1, and pcDNA-NC (Beina Biology, Beijing, China) were transfected or cotransfected into hypoxia-induced H9c2 cells using Lipofectamine 3000 (Invitrogen) for 48 h.

### 2.2. A Rat Model of MI

Animal experiments were approved by the local ethical committee in strict accordance with the Guide for the Care and Use of Laboratory Animals (No. KY012-18). Male wild-type Sprague-Dawley rats (SPF grade, 8 weeks, and 200-250 g) were from SPF Biotechnology (Beijing, China). The MI model was established in rats as previously described [32]. Simply, rats were intraperitoneal injected with 50 mg/kg pentobarbital sodium for anaesthesia, and then the left anterior descending artery (LAD) was ligated for 30 min (*n* = 30) [32]. A same procedure without LAD ligation was performed in rats of the sham group (*n* = 8). Before suturing the chest, 10 *μ*L pcDNA-SNHG4 and pcDNA-NC that packaged in adenovirus (5 × 10^10^ pfu/mL) were injected into the apex of the left ventricle with a 30-gauge needle (MI + Ad-SNHG4 and MI + Ad-NC groups; *n* = 10 each group). After the surgery for 4 weeks, the survival rate was 87.5%, 80%, 70%, and 80% in sham, MI, MI + Ad-SNHG4, and MI + Ad-NC groups, respectively. Six survival rats in each group were randomly selected for the following analyses. Transthoracic echocardiography was performed to detect the parameters of left ventricular fraction shortening (LVFS), left ventricular ejection fraction (LVEF), left ventricular end-diastolic diameter (LVEDd), and left ventricular end-systolic diameter (LVESd).

### 2.3. Triphenyltetrazolium Chloride (TTC) Staining

Followed by hemodynamic analyses, rats were anaesthetized with intraperitoneal injection of pentobarbital sodium (50 mg/kg) and were sacrificed via cervical dislocation. The heart was immediately resected, and the ventricle samples at a same depth were sliced into sections at 2 mm or 5 *μ*m. The sections were subsequently stained with 1% TTC for 15 min under darkness. After 6 h of fixing with 10% formaldehyde, the infarction area (white) was captured under a microscope (Olympus, Japan).

### 2.4. Histological Examinations

Some ventricle tissues were also collected for histological examinations. The ventricle tissues were fixed with 10% formaldehyde, paraffin-embedded, and sectioned into 5 *μ*m. After dewaxing in xylene and rehydration in gradient ethanol, the tissue sections were stained with hematoxylin-eosin (HE) (Sigma, San Luis, MO, USA), MASSON solution (Huzhen, Shanghai, China), or TUNEL (Thermo Fisher Scientific, Waltham, MA, USA). On the other hand, some sections were successively incubated with anti-Ki67 (1 : 1,500, Abcam, Cambridge, England), horseradish peroxidase- (HRP-) conjugated secondary antibody, and diaminobenzidine for immunohistochemistry (IHC). Followed by dehydration and vitrification, the sections were finally captured under a microscope (Olympus).

### 2.5. Quantitative Real-Time PCR (qRT-PCR)

Total RNAs were isolated from H9c2 cells or myocardial tissues using a RNA Extraction Kit (Promega, Madison, WI, USA) and then reverse transcribed into cDNAs using a cDNA Synthesis Kit (APExBio, Houston, TX, USA). Using a SYBR Green FAST Mastermix (Qiagen, Dusseldorf, Germany), qRT-PCR was performed on ABI7500 system (Applied Biosystems, Foster City, CA, USA). The mRNA expression of related genes was calculated according to the 2^−ΔΔct^ method and normalized to GAPDH (SNHG4 and DUSP1) or U6 (miR-148b-3p).

### 2.6. Cell Viability Assay

The viability of H9c2 cells was determined by MTT assay. Simply, 100 *μ*L H9c2 cells (1 × 10^5^ cells/mL) were cultured in 96-well plates for 48 h. After 2 h of incubation with 15 *μ*L MTT (Aladdin, Shanghai, China) at 37°C, the optical density at 450 nm (OD_450_) was measured by a microplate reader (Thermo Fisher Scientific).

### 2.7. Cell Apoptosis Assay

The caspase-3 in H9c2 cells was detected using a caspase-3 Assay Kit (QCbio, Shanghai, China) following the manufacturer's protocol. In addition, the apoptotic rate was measured using an annexin V-FITC Apoptosis Kit (Thermo Fisher Scientific). Briefly, 2 × 10^5^ cells that resuspended in 500 *μ*L binding buffer were incubated with 5 *μ*L annexin V-EGFP and 5 *μ*L PI for 15 min at 4°C under darkness. The apoptotic data was measured by a FACScan flow cytometer (BD, Franklin Lakes, NJ, USA).

### 2.8. Western Blot

H9c2 cells were lysed in RIPA buffer to isolate total proteins. After quantification using a BCA Assay Kit (Thermo Fisher Scientific), 30 *μ*g protein samples were run on 10% sodium dodecyl sulphate-polyacrylamide gel electrophoresis and transferred onto polyvinylidene fluoride membranes. The proteins in the membranes were then blocked with 5% skimmed milk and incubated with specific primary antibodies (anti-Bax, -Bcl-2, and -DUSP1; 1 : 1,500, Sigma) for 12 h at 4°C. Subsequently, the proteins were further incubated with HRP-conjugated secondary antibody (1 : 5,000, Sigma) for 1 h at 25°C. Using an ECL Kit (Thermo Fisher Scientific), the protein blots were visualized and captured under a Gel-Pro analyzer (Media Cybernetics, Silver Spring, MD, USA). *β*-Actin was an internal control for western blot (anti-*β*-actin, 1 : 2,000, Sigma).

### 2.9. Enzyme-Linked Immunosorbent Assay (ELISA)

The TNF-*α*, IL-1*β*, and IL-6 in hypoxia-induced H9c2 cells and in serum samples were measured using specific ELISA kits (enzyme-linked Biotechnology, Shanghai, China). In addition, the serum level of lactate dehydrogenase (LDH) was detected using a LDH Assay Kit (Abcam). All the above assays were performed following the manufacturers' protocols.

### 2.10. Dual Luciferase Reporter (DLR) Assay

The target relations between miR-148b-3p and SNHG4/DUSP1 were predicted by TargetScan [33] and/or StarBase [34]. For DLR assay, the wild type (wt) and mutant (mut) SNHG4/DUSP1 sequences containing miR-148b-3p binding site were integrated into pGL3 vector (Promega), named as SNHG4-wt/DUSP1-wt and SNHG4-mut/DUSP1-mut. H9c2 cells were cotransfected with these recombinant vectors and miR-148b-3p mimics/miR-NC for 48 h. The luciferase activity was detected using a DLR Assay System (Promega).

### 2.11. Statistical Analyses

The experiments *in vitro* were performed in triplicate, and those *in vivo* were performed in six rats of each group, respectively. Data that expressed as mean ± standard deviation (SD) were statistically analyzed using SPSS 23.0. The comparison between two groups was analyzed by Student's *t*-test and that among multiple groups was analyzed by one-way ANOVA with Tukey's test. *P*value < 0.05 presented significantly different.

## 3. Results

### 3.1. SNHG4 Overexpression Enhances the Viability and Represses the Apoptosis and Inflammation of Hypoxia-Induced H9c2

**Figure 1:**
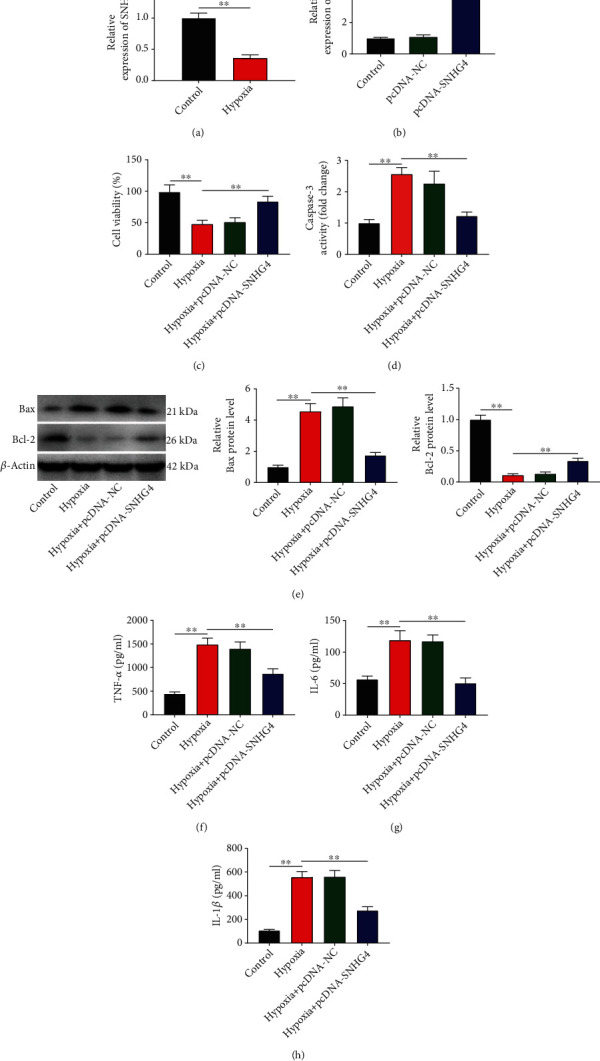
Overexpression of SNHG4 promotes the viability and represses the apoptosis and inflammation of hypoxia-induced H9c2 cells. (a) The expression of SNHG4 in hypoxia-induced H9c2 cells was detected by qRT-PCR. (b) The expression of SNHG4 in hypoxia-induced H9c2 cells transfected with pcDNA-SNHG4/pcDNA-NC was detected by qRT-PCR. (c) Cell viability (OD450) was measured by MTT assay. (d) Caspase-3 activity. (e) The protein expression of Bax and Bcl-2 were measured by western blot. (f–h) The levels of TNF-*α*, IL-6, and IL-1*β* were measured by ELISA. ^∗∗^*P* < 0.01.

In hypoxia-induced H9c2 cells, a significantly lower expression of SNHG4 was revealed than in the controls (*P* < 0.01; Figure 1(a)). Given this, SNHG4 was overexpressed to investigate the function of SNHG4. In comparison with the control cells, SNHG4 was upregulated by pcDNA-SNHG4 (*P* < 0.01) but not influenced by pcDNA-NC (Figure 1(b)). Subsequently, cellular function assays were performed. Hypoxia-treated H9c2 cells exhibited significantly decreased cell viability, increased caspase-3 activity, upregulated Bax, downregulated Bcl-2, and elevated TNF-*α*/IL-1*β*/IL-6 than in the controls (*P* < 0.01; Figures 1(c)–1(h)). The transfection of pcDNA-SNHG4 weakened the effects of hypoxia on inhibiting cell viability and on promoting apoptosis (caspase-3/Bax/Bcl-2) and inflammation (TNF-*α*/IL-1*β*/IL-6) (hypoxia vs. hypoxia + pcDNA-SNHG4, *P* < 0.01; Figures 1(c)–1(h)).

### 3.2. miR-148b-3p is an Action Target of SNHG4

**Figure 2:**
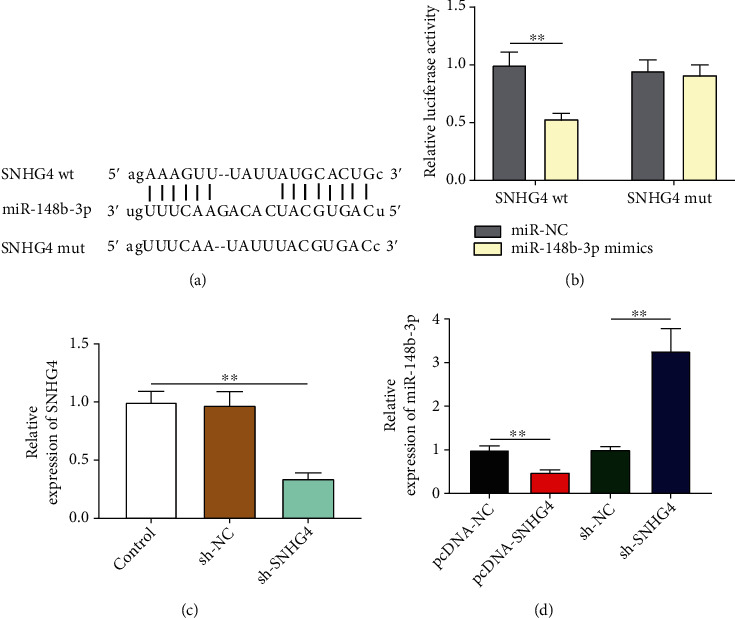
The target relationship between miR-148b-3p and SNHG4. (a) A binding site of SNHG4 and miR-148b-3p was predicted by StarBase. (b) DLR assay of the target relationship between miR-148b-3p and SNHG4. (c) The expression of SNHG4 in H9c2 cells transfected with sh-SNHG4/sh-NC was detected by qRT-PCR. (d) The expression of miR-148b-3p in H9c2 cells transfected with pcDNA-SNHG4/pcDNA-NC/sh-SNHG4/sh-NC was detected by qRT-PCR. ^∗∗^*P* < 0.01.

StarBase predicted the presence of a binding site between SNHG4 and miR-148b-3p (Figure 2(a)). This target relation was then confirmed by DLR assay, evidenced by a lower luciferase activity of SNHG4 wt-transfected cells in the miR-148b-3p mimic group than in the miR-NC group (*P* < 0.01; Figure 2(b)). SNHG4 was further silenced by the transfection of sh-SNHG4 (control vs. sh-SNHG4, *P* < 0.01; Figure 2(c)). qRT-PCR determined a significantly decreased miR-148b-3p expression in the pcDNA-SNHG4 group than in the pcDNA-NC group (*P* < 0.01). On the contrary, the sh-SNHG4 group showed a higher expression of miR-148b-3p than in the sh-NC group (*P* < 0.01; Figure 2(d)).

### 3.3. Silencing of miR-148b-3p Enhances the Viability and Represses the Apoptosis and Inflammation of Hypoxia-Induced H9c2

**Figure 3:**
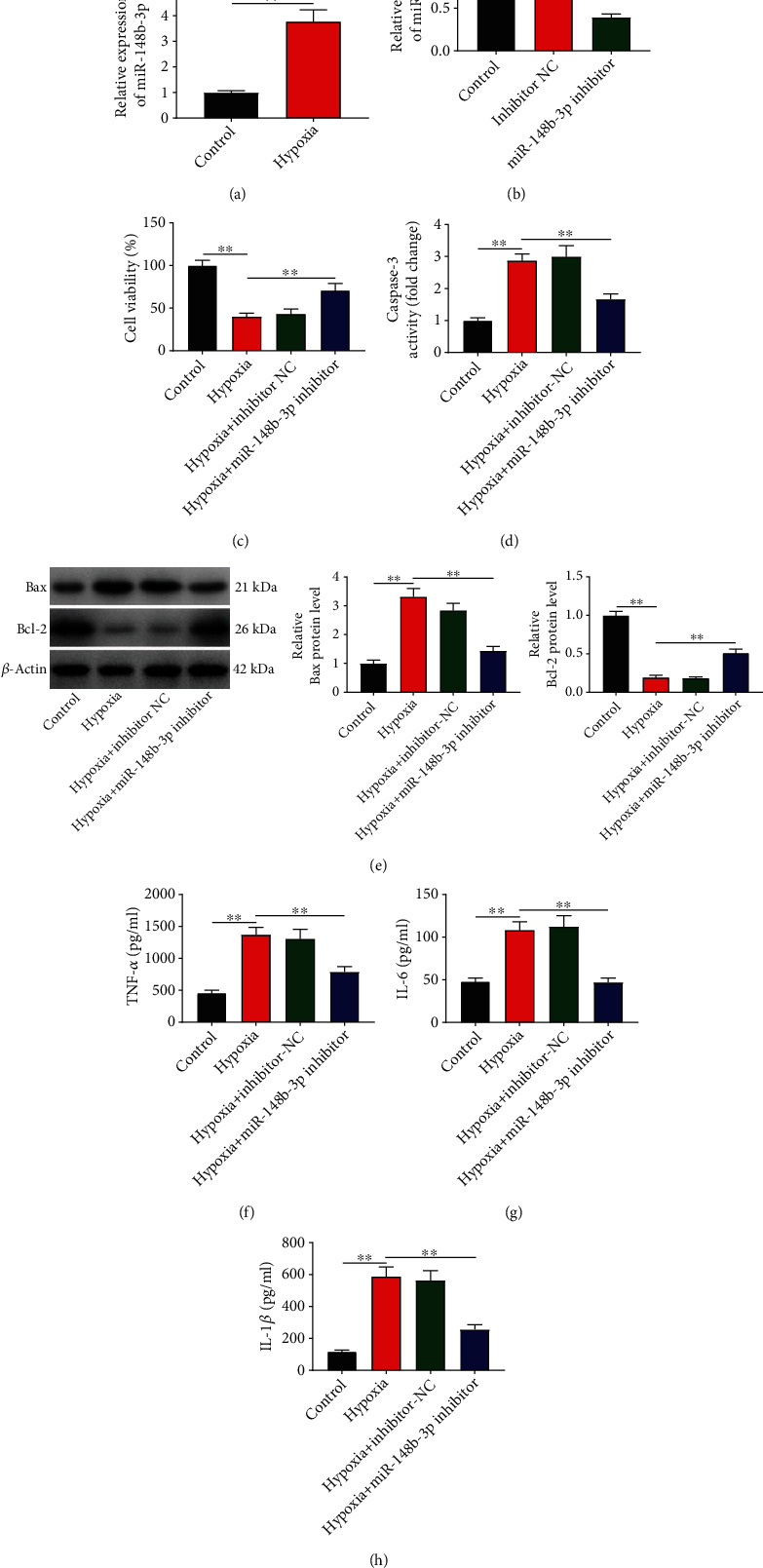
Silencing of miR-148b-3p promotes the viability and represses the apoptosis and inflammation of hypoxia-induced H9c2 cells. (a) The expression of miR-148b-3p in hypoxia-induced H9c2 cells was detected by qRT-PCR. (b) The expression of miR-148b-3p in hypoxia-induced H9c2 cells transfected with miR-148b-3p inhibitor/inhibitor-NC was detected by qRT-PCR. (c) Cell viability (OD450) was measured by MTT assay. (d) Caspase-3 activity. (e) The protein expression of Bax and Bcl-2 were measured by western blot. (f–h) The levels of TNF-*α*, IL-6, and IL-1*β* were measured by ELISA. ^∗∗^*P* < 0.01.

The function of miR-148b-3p in MI model *in vitro* was subsequently evaluated. qRT-PCR revealed a higher miR-148b-3p expression in hypoxia-induced H9c2 cells than in the controls (*P* < 0.01; Figure 3(a)). miR-148b-3p inhibitor effectively silenced miR-148b-3p in H9c2 cells (control vs. miR-148b-3p inhibitor, *P* < 0.01; Figure 3(b)). The following functional assays determined enhanced cell viability, decreased caspase-3 activity, downregulated Bax, upregulated Bcl-2, and reduced TNF-*α*/IL-1*β*/IL-6 in the hypoxia + miR-148b-3p inhibitor group in comparison with the hypoxia group (*P* < 0.01; Figures 3(c)–3(h)).

### 3.4. DUSP1, a Target of miR-148b-3p, Represses the Apoptosis of Hypoxia-Induced H9c2

**Figure 4:**
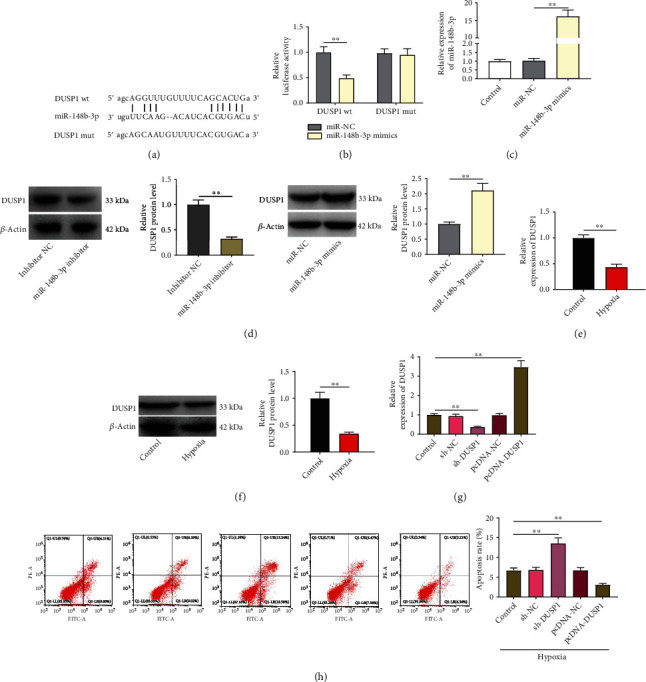
The target relationship between DUSP1 and miR-148b-3p. (a) A binding site of DUSP1 and miR-148b-3p was predicted by TargetScan and StarBase. (b) DLR assay of the target relationship between DUSP1 and miR-148b-3p. (c) The expression of miR-148b-3p in H9c2 cells transfected with miR-148b-3p mimics/miR-NC was detected by qRT-PCR. (d) The protein expression of DUSP1 in H9c2 cells transfected with miR-148b-3p inhibitor/inhibitor NC or miR-148b-3p mimics/miR-NC was detected by western blot. (e) The mRNA expression of DUSP1 in hypoxia-induced H9c2 cells was detected by qRT-PCR. (f) The protein expression of DUSP1 in hypoxia-induced H9c2 cells was detected by western blot. (g) The mRNA expression of DUSP1 in hypoxia-induced H9c2 cells transfected with sh-DUSP1/sh-NC/pcDNA-NC/pcDNA-DUSP1 was detected by qRT-PCR. (h) Cell apoptosis was detected by flow cytometry. ^∗∗^*P* < 0.01.

As shown in Figure 4(a), both TargetScan and StarBase predicted a binding site between miR-148b-3p and DUSP1. DLR assay revealed a lower luciferase activity of DUSP1 wt-transfected cells in the miR-148b-3p mimic group than the miR-NC group (*P* < 0.01; Figure 4(b)), which confirmed the target relationship. In addition, there were significantly higher miR-148b-3p and DUSP1 expression in the miR-148b-3p mimic group than in the miR-NC group (*P* < 0.01) and significantly lower DUSP1 expression in the miR-148b-3p inhibitor group than in the inhibitor NC group (*P* < 0.01; Figures 4(c) and 4(d)). Besides, both the mRNA and protein expression of DUSP1 were lower in hypoxia-induced H9c2 cells than in the controls (*P* < 0.01; Figures 4(e) and 4(f)). Since DUSP is an antiapoptotic phosphatase, the regulatory role of DUSP1 on cell apoptosis was further explored. DUSP1 was silenced and overexpressed by the transfection of sh-DUSP1 and pcDNA-DUSP1 in H9c2 cells, respectively (sh-DUSP1 and pcDNA-DUSP1 vs. control, *P* < 0.01; Figure 4(g)). When compared with the controls, the apoptotic rate of hypoxia-induced H9c2 cells was enhanced by DUSP1 silencing but inhibited by DUSP1 overexpression (*P* < 0.01; Figure 4(h)).

### 3.5. The Regulatory Mechanisms of SNHG4/miR-148b-3p/DUSP1 in Hypoxia-Induced H9c2

**Figure 5:**
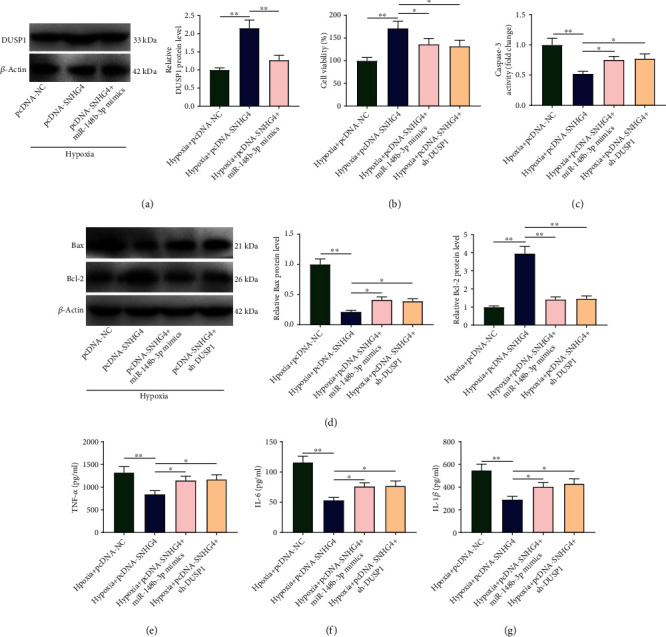
The regulatory mechanisms of SNHG4 involving miR-148b-3p/DUSP1 in hypoxia-induced H9c2 cells. (a) The protein expression of DUSP1 in hypoxia-induced H9c2 cells was determined by western blot. (b) Cell viability (OD450) was measured by MTT assay. (c) Caspase-3 activity. (d) The protein expression of Bax and Bcl-2 were measured by western blot. (e–g) The levels of TNF-*α*, IL-6, and IL-1*β* were measured by ELISA. ^∗^*P* < 0.05; ^∗∗^*P* < 0.01.

The action mechanisms of SNHG4/miR-148b-3p/DUSP1 axis were verified in hypoxia-induced H9c2. As a result, miR-148b-3p mimics decreased the upregulation of DUSP1 in hypoxia + pcDNA-SNHG4 group (hypoxia +pcDNA-SNHG4+miR-148b-3p mimics vs. hypoxia + pcDNA-SNHG4, *P* < 0.01; Figure 5(a)). In comparison with the hypoxia + pcDNA-SNHG4 group, the hypoxia + pcDNA-SNHG4 + miR-148b-3p mimic group exhibited significantly decreased cell viability, increased caspase-3 activity, upregulated Bax, downregulated Bcl-2, and elevated TNF-*α*/IL-1*β*/IL-6 (*P* < 0.05; Figures 5(b)–5(g)). Similarly with miR-148b-3p mimics, sh-DUSP1 also significantly weakened the effects of pcDNA-SNHG4 on enhancing cell viability and on inhibiting apoptosis and inflammation (hypoxia + pcDNA-SNHG4 vs. hypoxia + pcDNA-SNHG4 + sh-DUSP1, *P* < 0.05; Figures 5(b)–5(g)).

### 3.6. The Expression of SNHG4, miR-148B-3p, and DUSP1 in MI Rats

**Figure 6:**
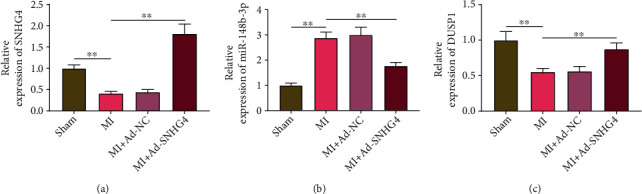
The expression of (a) SNHG4, (b) miR-148B-3p, and (c) DUSP1 in MI rats was detected by qRT-PCR. ^∗∗^*P* < 0.01.

To identify the role of SNHG4/miR-148b-3p/DUSP1 axis *in vivo*, a rat model of MI was established. MI rats showed lower expression of SNHG4 and DUSP1 and higher expression of miR-148b-3p than in the sham rats (*P* < 0.01). The injection of ad-SNHG4 reversed the downregulation of SNHG4 and DUSP1 and upregulation of miR-148b-3p in MI rats (MI vs. MI + Ad-SNHG4, *P* < 0.01, Figures 6(a)–6(c)).

### 3.7. SNHG4 Overexpression Relieves the Myocardial Injury in MI Rats

**Figure 7:**
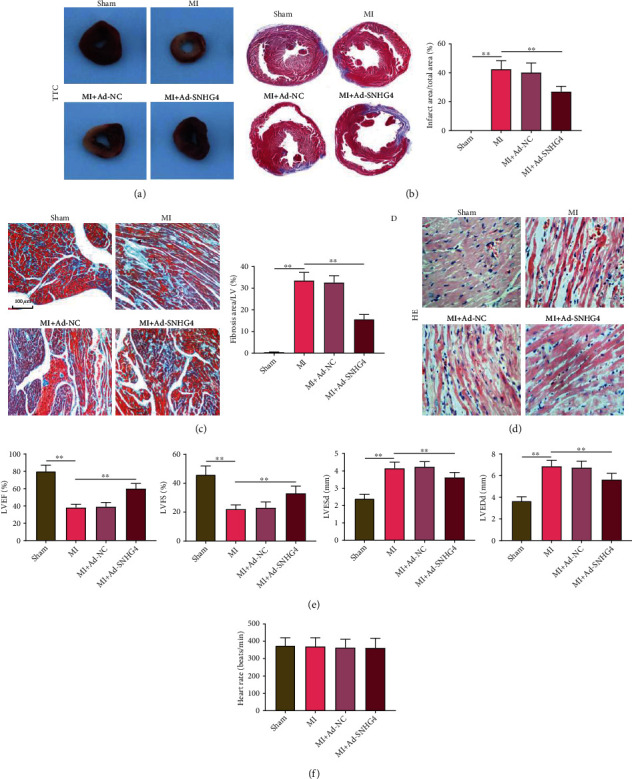
The effects of SNHG4 overexpression on myocardial injury in MI rats. (a, b) The infarct area was observed by TTC staining (×200). (c) The fibrosis area was observed by MASSON's staining (×200). (d) The pathological changes were observed by HE staining (×200). (e) The levels of LVEF, LVFS, LVESd, and LVEDd were measured by echocardiography. (f) The heart rate in rats. ^∗∗^*P* < 0.01.

The effects of SNHG4 overexpression on the myocardial injury of MI rats were evaluated. TTC staining showed an obvious reduction of infarct area in rats of Ad-SNHG4 + MI group than in the MI group (*P* < 0.01; Figures 7(a)–7(b)). Overexpression of SNHG4 also significantly reduced fibrosis area in MI rats (Ad-SNHG4 + MI vs. MI, *P* < 0.01; Figure 7(c)). HE staining revealed swelling cardiomyocytes, irregular arranged nucleus, interstitial enlargement, and neutrophil infiltration in MI rats. These pathological changes were partially relieved in MI rats injected with ad-SNHG4 (Figure 7(d)). Furthermore, hemodynamic analyses uncovered lower LVEF and LVFS and higher LVESd and LVEDd in the MI group than in the sham group (*P* < 0.01), and these changes were all weakened by the injection of ad-SNHG4 (MI + Ad-SNHG4 vs. MI, *P* < 0.01; Figure 7(e)). However, no significantly differences on the heart rate were revealed among different groups (Figure 7(f)).

### 3.8. SNHG4 Overexpression Inhibits Cell Apoptosis and Inflammation in MI Rats

**Figure 8:**
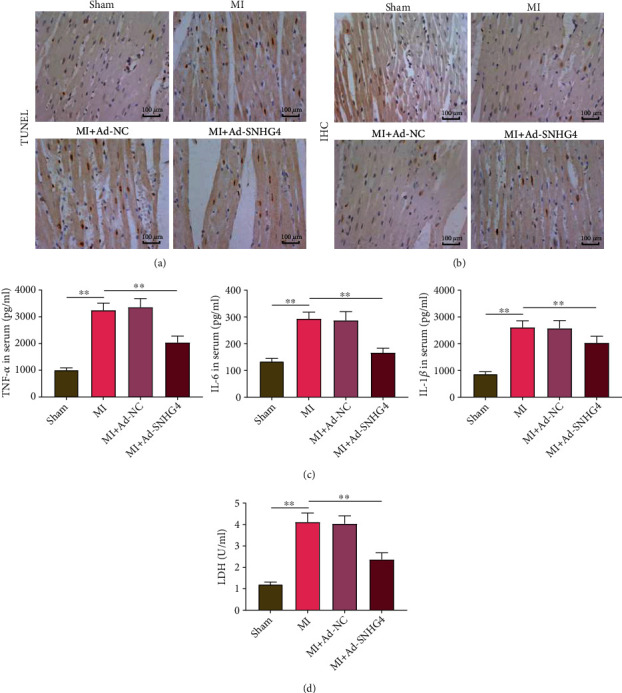
The effects of SNHG4 overexpression on the apoptosis and inflammation of myocardial cells in MI rats. (a) The apoptotic cells were observed by TUNEL staining. (b) Ki67-positive cells were observed by IHC. (c) The serum levels of TNF-*α*, IL-6, and IL-1*β* were measured by ELISA. (d) The serum level of LDH. ^∗∗^*P* < 0.01.

In MI rats, cell apoptosis and inflammation in myocardial tissues were further explored. TUNEL assay showed that there are more apoptotic cells in the MI group than in the sham group and less apoptotic cells in the MI + Ad-SNHG4 group than in the MI group (Figure 8(a)). On the contrary, IHC determined that there are less Ki67-positive cells in the MI group than in the sham group and more Ki-67-positive cells in the MI + Ad-SNHG4 group than in the MI group (Figure 8(b)). In addition, the serum TNF-*α*, IL-6, IL-1*β*, and LDH were higher in MI rats than in the sham rats (*P* < 0.01). The injection of ad-SNHG4 inhibited the increasing of the above parameters in MI rats (MI + Ad-SNHG4 vs. MI, *P* < 0.01; Figures 8(c) and 8(d)).

## 4. Discussion

MI is a life-threatening cardiovascular disorder that characterized by myocardial necrosis on account of acute ischemia and hypoxia [26]. With the increasing of healthcare costs in patients with MI [35], exploring effective molecular targets is urgently helpful for the treatment MI. Until now, the diverse lncRNAs are abnormally expressed in MI. For examples, ANRIL [36], GAS5 [23], and KCNQ1OT1 [37] are upregulated; whereas, SLC8A1-AS1 [38], H19 [5], and Gm2619 [4] are downregulated in cell or animal models of MI. In our study, SNHG4 was significantly downregulated in hypoxia-treated H9c2 cells than in the controls and also in MI rats than in the sham rats. This result heralds a potential role of SNHG4 in the pathogenesis of MI.

lncRNAs exert critical roles in MI through regulating various cellular processes, mainly including cell viability, apoptosis, and inflammation [8, 10, 11]. For example, overexpression of H19 represses the development of MI via inhibiting inflammatory reaction and apoptosis in myocardial cells [5]. The upregulation of Gm2691 ameliorates cardiac function through suppressing myocardial fibrosis, apoptosis, and inflammation [4]. The upregulation of CARL increases myocardial cell viability and decreases cell apoptosis in MI rats [3]. Here, researches on the regulatory role of SNHG4 in MI showed that overexpression of SNHG4 inhibited cell apoptosis and inflammation and enhanced cell viability in both hypoxia-treated H9c2 cells and MI rats (hypoxia + pcDNA-SNHG4 vs. hypoxia, MI + Ad-SNHG4 vs. MI). These results indicate a protective role of SNHG4 in myocardial cells under MI, which are similar with those reported lncRNAs mentioned above. In addition, SNHG4 overexpression also decreased infarct and fibrosis areas, relieved histopathological damages, and improved heart function in MI rats (MI + Ad-SNHG4 vs. MI). These findings further confirm the therapeutic potential of SNHG4 against MI. To sum up, SNHG4 may relieve MI through inhibiting the apoptosis, inflammation, and fibrosis of myocardial cells.

lncRNAs are widely known as sponges for miRNAs, and this interaction has been studied throughout physiological and disease states [39]. In this study, the potential miRNA target of SNHG4 was explored to determine the action mechanisms of SNHG4 in MI. A target relation between SNHG4 and miR-148b-3p was predicted, which was subsequently confirmed by DLR assay. With the ability to regulate cell proliferation, apoptosis, migration, and invasion, miR-148b-3p acts an antioncogene in different human cancers, such as gastric cancer [40], gastrointestinal stromal tumor [41], hepatocellular carcinoma [42], and renal carcinoma [43]. miR-148b-3p also participates in the pathogenesis of cardiovascular disorders. Chen et al. have found that the miR-148b-3p in the blood is a diagnostic marker for ischemic stroke [44]. Wang et al. have revealed that miR-148b-3p is involved in inhibiting neural stem cell proliferation and differentiation, and its silencing relieves ischemic lesion in a rat model of ischemic stroke [45]. Notably, the downregulation of miR-148b ameliorates I/R-induced cardiac dysfunction and myocardial infarction and increases the viability of myocardial cells [20]. In this research, miR-148b-3p was upregulated in hypoxia-treated H9c2 cells compared with controls. Silencing of miR-148b-3p in hypoxia-treated H9c2 cells inhibited cell apoptosis and inflammation and promoted cell viability (hypoxia + miR-148b-3p inhibitor vs. hypoxia). These findings are similar with previous studies and indicate that miR-148b-3p inhibitor may protect myocardial cells against hypoxia-induced injury. To combine with the negative regulatory relation between SNHG4 and miR-148b-3p, we suspect that miR-148b-3p downregulation may contribute to the relieving role of SNHG4 on MI. Furthermore, our following feedback assays determined that miR-148b-3p overexpression eliminated the protective effects of SNHG4 overexpression on hypoxia-treated H9c2 cells, evidenced by weakened cell viability, as well as enhanced cell apoptosis and inflammation (hypoxia + pcDNA-SNHG4 vs. hypoxia + pcDNA-SNHG4 + miR-148b-3p mimics). Therefore, we believe that SNHG4 may attenuate the myocardial injury of MI by targeting miR-148b-3p.

Since lncRNAs and miRNAs are both noncoding RNAs, their functions rely on the translation or degradation of target mRNAs [46]. To further reveal a complete regulatory axis of lncRNA/miRNA/mRNA in MI, the mRNA target of miR-148b-3p was investigated. The results determined that DUSP1 was a downstream target of miR-148b-3p. As an antiapoptotic phosphatase, DUSP1 can act as an oncogene in gastric cancer [47], breast cancer [23], and osteosarcoma [48]. DUSP1 also exerts a critical regulatory role in myocardial I/R injury. He et al. have shown that the downregulation of TRIM11 inhibits the apoptosis of cardiomyocytes following I/R injury through regulating DUSP1-JNK1/2 pathway [49]. Ren et al. have revealed that DUSP1 enhances the antiapoptotic effect of NaHS in rats with myocardial I/R injury [50]. Jin et al. have revealed that DUSP1 alleviates myocardial I/R injury through inhibiting mitochondrial fission and mitophagy [51]. Therefore, the upregulation of DUSP1 may also be a benefit for the remission of MI via similar mechanisms mentioned above. Since DUSP1 can be negatively regulated by miR-148b-3p and positively regulated by SNHG4, SNHG4 may protect myocardial cells against MI injury through regulating miR-148b-3p/DUSP1 axis. As expected, DUSP1 silencing in hypoxia-treated H9c2 weakened the enhancing effect of SNHG4 overexpression on cell viability and the inhibiting effects on cell apoptosis and inflammation (hypoxia + pcDNA-SNHG4 vs. hypoxia + pcDNA-SNHG4+sh-DUSP1). These findings further illustrate the presence of SNHG4/miR-148b-3p/DUSP1 axis in MI.

In fact, there are many downstream and upstream regulators of the SNHG4/miR-148b-3p/DUSP1 axis that are involved in MI. Evidence has determined that lncRNAs can be regulated by many core transcription factors, such as SP1, NF-*κ*B, p53, and SOX2 [52]. These transcription factors also play an important role in regulating myocardial injury [53–56]. In addition, the protective role of DUSP1 in myocardial injury has been determined to be closely associated the downstream pathways of JNK [51, 57] and MAPK [58]. These regulators may participate in the regulatory mechanisms of the SNHG4/miR-148b-3p/DUSP1 axis in MI.

This study also exhibits some limitations. For examples, the action mechanisms of SNHG4/miR-148b-3p/DUSP1 axis are not confirmed in MI rats. The downstream and upstream mechanisms of SNHG4/miR-148b-3p/DUSP1 axis in MI need further exploration. Besides, miR-148b-3p/DUSP1 is not the only downstream target of SNHG4 in MI. These limitations still need to be solved by future researches.

## 5. Conclusions

In conclusion, SNHG4 and DUSP1 were downregulated, and miR-148b-3p was upregulated in both cellular and rat models of MI. Overexpression of SNHG4 promoted the viability and repressed the apoptosis and inflammation of hypoxia-induced H9c2 cells through regulating miR-148b-3p/DUSP1. In addition, SNHG4 overexpression also relieved myocardial injury in MI rats. Our findings illustrate an important regulatory axis of SNHG4/miR-148b-3p/DUSP1 in MI, providing potential therapeutic targets.

## Data Availability

All data in the manuscript are available through the responsible corresponding author.
